# Effects of amyloid β (Aβ)42 and Gasdermin D on the progression of Alzheimer’s disease *in vitro* and *in vivo* through the regulation of astrocyte pyroptosis

**DOI:** 10.18632/aging.205174

**Published:** 2023-11-02

**Authors:** Wenjuan Hong, Chengping Hu, Can Wang, Binggen Zhu, Ming Tian, Hongyun Qin

**Affiliations:** 1 Department of Psychiatry, Shanghai Pudong New Area Mental Health Center, Tongji University School of Medicine, Shanghai 200124, China; 2 Department of Burn, Wound Healing Center, Ruijin Hospital, Shanghai Jiaotong University School of Medicine, Shanghai 200025, China

**Keywords:** astrocyte pyroptosis, endothelial cell, GSDMD, Alzheimer’s disease, amyloid β

## Abstract

Purpose: The study aimed to investigate whether astrocyte pyroptosis, and the subsequent neuroinflammatory response that exerts amyloid β (Aβ) neurotoxic effects, has an effect on endothelial cells, along with the underlying mechanisms.

Methods: *In vivo*, 5 μL of disease venom was injected into the lateral ventricle of APP/PS1 mice for treatment. Pyroptosis was induced by treating astrocytes with Aβ42 *in vitro*. Small interfering RNA (siRNA) was used to silence caspase-1 and Gasdermin D (GSDMD) mRNA expression. Cell viability was determined using a CCK-8 detection kit. Scanning electron microscopy (SEM), Annexin V/propidium iodide (PI) double staining, RT-qPCR, immunofluorescence, western blotting, and enzyme-linked immunosorbent assay (ELISA) were used to detect cell pyroptosis. The degree of pathological damage to the brain and aortic tissue was assessed by hematoxylin-eosin staining and immunohistochemistry.

Results: Aβ42 induced astrocyte pyroptosis dependent on the GSDMD/Gasdermin E (GSDME)/Caspase 11/NLRP3 pathway, releasing large amounts of inflammatory factors, such as TNF-α, IL-1α, IL-1β, and IL-18. Astrocyte pyroptosis caused endothelial cell dysfunction and release of large amounts of vasoconstrictors (ET and vWF). Knockdown of GSDMD reduced astrocyte pyroptosis in the cerebral cortex and hippocampal tissue, decreased the release of inflammatory factors IL-1 β and IL-18, reduced Aβ deposition and tau protein, increased the release of peripheral vasodilator substances (eNOS), and decreased the release of vasoconstrictor substances (ET, vWF), thereby reducing brain tissue damage and vascular injury in APP/PS1 mice.

Conclusion: Aβ42 induced astrocyte pyroptosis, while GSDMD knockout inhibited astrocyte pyroptosis, reduced the release of inflammatory factors, and alleviated brain tissue damage and vascular damage in APP/PS1 mice. Therefore, GSDMD is a novel therapeutic target for Alzheimer’s disease.

Purpose: The study aimed to investigate whether astrocyte pyroptosis, and the subsequent neuroinflammatory response that exerts amyloid β (Aβ) neurotoxic effects, has an effect on endothelial cells, along with the underlying mechanisms.

Methods: *In vivo*, 5 μL of disease venom was injected into the lateral ventricle of APP/PS1 mice for treatment. Pyroptosis was induced by treating astrocytes with Aβ42 *in vitro*. Small interfering RNA (siRNA) was used to silence caspase-1 and Gasdermin D (GSDMD) mRNA expression. Cell viability was determined using a CCK-8 detection kit. Scanning electron microscopy (SEM), Annexin V/propidium iodide (PI) double staining, RT-qPCR, immunofluorescence, western blotting, and enzyme-linked immunosorbent assay (ELISA) were used to detect cell pyroptosis. The degree of pathological damage to the brain and aortic tissue was assessed by hematoxylin-eosin staining and immunohistochemistry.

Results: Aβ42 induced astrocyte pyroptosis dependent on the GSDMD/Gasdermin E (GSDME)/Caspase 11/NLRP3 pathway, releasing large amounts of inflammatory factors, such as TNF-α, IL-1α, IL-1β, and IL-18. Astrocyte pyroptosis caused endothelial cell dysfunction and release of large amounts of vasoconstrictors (ET and vWF). Knockdown of GSDMD reduced astrocyte pyroptosis in the cerebral cortex and hippocampal tissue, decreased the release of inflammatory factors IL-1 β and IL-18, reduced Aβ deposition and tau protein, increased the release of peripheral vasodilator substances (eNOS), and decreased the release of vasoconstrictor substances (ET, vWF), thereby reducing brain tissue damage and vascular injury in APP/PS1 mice.

Conclusion: Aβ42 induced astrocyte pyroptosis, while GSDMD knockout inhibited astrocyte pyroptosis, reduced the release of inflammatory factors, and alleviated brain tissue damage and vascular damage in APP/PS1 mice. Therefore, GSDMD is a novel therapeutic target for Alzheimer’s disease.

## INTRODUCTION

Alzheimer’s disease (AD) is a degenerative disorder of the central nervous system (CNS) that seriously affects the health of older people. Despite the elusive mechanisms underlying the pathogenesis of AD, considerable evidence suggests that multiple factors are involved in AD, including amyloid β (Aβ) plaque, neurofibrillary tangles (NFTs), synaptic dysfunction and neurotransmitter imbalance, oxidative stress, and neuroinflammation [1]. Inflammation has been strongly implicated in the pathogenesis of AD during aging [2–5]. Neuroinflammation is closely linked to peripheral immune activation, and cognitive decline is associated with increased peripheral inflammation, and pro-inflammatory cytokines, such as interleukin-1β (IL-1β), interleukin-6 (IL-6) and tumor necrosis factor-α (TNF-α) [6]. The Dietary Approaches to Stop Hypertension (DASH), which have hypotensive and anti-inflammatory effects, are associated with reduced cognitive decline [2, 3]. Evidence showed that inflammation-induced dysfunctions of endothelial cells (EC) have an effect on hypertension [7], and hypertension-related chronic cerebral hypofusion increases the deposition of Aβ [8]. The dysfunctions of EC are probably associated with cognitive decline.

Pyroptosis, a kind of inflammatory programmed cell death, is characterized by cellular swelling, membrane pore formation, plasma membrane rupture, assembly of inflammasomes, and release of a large amount of IL-1β and interleukin 18 (IL-18) [9]. Inflammasomes, key components of the natural immune system, play an important role in the immune response and pathogenesis of numerous CNS (central nervous system) diseases, including AD [10–12]. Studies have also shown glial cell aggregation in the brain tissue of patients and AD rats, causing inflammation, and abnormally elevated expression of inflammatory factors, which further amplifies the inflammatory cascade. Astrocytes (AS), one of the neuroglials, regulate CNS homeostasis by synchronizing neuronal activity [13, 14]. However, under aberrant amyloid plaque accumulation conditions, they become reactive astrocytes, promoting Aβ pathology by producing inflammation and NLRP3 inflammasome priming/activation, which may be responsible for cognitive impairment in the whole process of AD [15–17]. In view of the role of pyroptosis in the inflammatory response, this study investigated the effect of Aβ on astrocytes and its molecular mechanism, in order to further elucidate the role of astrocyte pyroptosis in AD-related inflammatory response and its effect on endothelial cells.

## MATERIALS AND METHODS

### Cell culture

Astrocytes were obtained from the cerebral cortex of newborn mice (5 days old). Briefly, the neonatal mouse cortex was isolated and digested with 0.25% trypsin-ethylene diamine tetraacetic acid (EDTA) at 37°C for 30 minutes. After centrifugation at 300 rpm for 5 min, purified astrocytes cultured in DMEM containing 10% fetal bovine serum (FBS) in an incubator at 37°C and 5% CO_2_. Astrocytes were grouped when they reached approximately 80%. Astrocytes were routinely cultured in the control, 0.1 μmoL Aβ42 and 1 μmoL Aβ42 groups. The human Aβ42 (β-amyloid polypeptide 1–42, cat no. ab120301) was purchased from Abcam (Cambridge, UK).

### Intervention and grouping of APP/PS1 mice

Six-week-old APPswe/PS1dE9 double transgenic mice (B6C3-Tg (APPswe, PSEN1dE9), 85Dbo/Mmjax) were purchased from the Jackson Laboratory (004462). APP/PS1 mice were double transgenic, with β-amyloid deposition beginning in the brain at 6–7 months of age. In our study, 7-month-old mice were used, seven mice without APP/PS1 gene (Wild-Type Mice) were used as control group, and 15 APP/PS1 mice were divided into NC and shRNA-GSDMD groups. Briefly, mice were anesthetized by intraperitoneal injection of 20 mg/mL sodium pentobarbital solution. The hair on the top of the skull of each mouse was shaved, and the scalp was cut open. The skull surface was wiped with 30% hydrogen peroxide to expose the skull fontanelle and cranial sutures fully. A microsampler was used to inject 5 μL of disease venom (pAAV-GfaABC1D-EGFP-3xFLAG-miR30shRNA (Gsdmd) -WPRE) into the lateral ventricle of mice. In addition, the same dose of control carrier solution (pAAV-short GFAP-MCS-EGFP-3FLAG) was injected into the NC group mice. The adenovirus-associated vector of GSDMD was synthesized by Heyuan Biotechnology (Shanghai) Co., Ltd., China

All mice were housed under a 12/12 h inverted light/dark cycle (light switched on at 8:00), with a room temperature between 23–25°C, and ad libitum access to food and water. The experimental protocol was approved by the Experimental Animal Care and Use Committee of Tongji University School of Medicine (TJLA-020-118).

### siRNA transfection

GSDMD siRNA or caspase1 siRNA or scramble siRNA (30 μM) was diluted in DEPC to 20 μmol/L. The siRNA complex suspension was incubated at room temperature for 20 min and added to a 2 × 106 well cell-culture plate seeded with astrocytes. Forty-eight hours after transfection, the GSDMD knockdown efficiency was analyzed, and the positive staining rate was ≥80% for use. All siRNA primers (Table 1) were designed and synthesized by GenePhrama (Shanghai, China).

**Table 1:** Primer sequence of siRNA.

**Gene**	**Primer sequence**
Caspase1	GGGCAAAGAGGAAGCAATTTA
Caspase11	CTTGTCATCTCTTTGATATAT
GSDME	CCGTCAAGAGAACAGTTAATA
GSDMD	GATTGATGAGGAGGAATTAAT
NLRP3	CCAGGAGAGAACCTCTTATTT

### Cell co-culture

To observe the effect of astrocytes in the scorched state on the secretory function of vascular endothelial cells, mouse astrocytes and aortic vascular endothelial cells were co-cultured. Mouse aortic endothelial cells were purchased from iCell Bioscience Inc., Shanghai, China. The co-culture system comprises of lower and upper chambers separated by a selectively permeable membrane with 0.4 μm-diameter pores. In the Transwell system, donor cells (vascular endothelial cells) were inoculated into polycarbonate membranes placed on top of recipient cells and co-cultured for 48 h. The experiment was divided into six groups, according to different treatment methods, as follow: group one, EC cells only; group two, EC cells co-cultivate with AS; group three, EC cells with Aβ42 (1 μM); group four, EC cells co-cultivate with AS with Aβ42 (1 μM); group five, EC cells co-cultivate with scramble transfected AS with Aβ42 (1 μM); and group six, EC cells co-cultivate with siGSDMD transfected AS with Aβ42 (1 μM).

### Cell counting Kit-8 (CCK-8) assay

The CCK-8 (CK04, Dojindo Molecular Technologies, Inc, Kumamoto, Japan) was used to assess cell viability. Astrocytes were seeded in 96-well plates at 5 × 10^4^ cells/well. After a 24 h culture, 10 μL of CCK-8 solution was added to each well and further cultured at 37°C for 2 h. Then, the absorbance at 450 nm was measured by a microplate reader (Multiskan FC, Thermo Fisher).

### Scanning electron microscopy (SEM) observation

Astrocytes were seeded in 96-well plates and co-cultured with or without GO (1 μm) for 24 h. Then, the astrocytes were fixed for 2 h with 2.5% glutaraldehyde at 4°C, and washed with 0.1 M phosphate buffer (pH = 7.4). Thereafter, the cells obtained were fixed in 1% osmic acid in 0.1 M phosphate buffer (pH = 7.4) for 1 h. Finally, graded ethanol was used to dehydrate the samples. A SEM was used to examine and photograph the cells (SU8100, Hitachi).

### Pyroptosis assay

Pyroptosis was detected by flow cytometry with Annexin-FITC/PI. The cells were collected and washed twice with cold PBS. The cells were then re-suspended with 100 μL PBS, after which 5 μL AnnexinV and 5 μL propidium iodide (PI) were mixed in evenly, and the mixture was incubated in the dark for 15 min. The cells were then assessed by flow cytometry (Beckman; Palo Alto, CA, USA).

### Real-time quantitative polymerase chain reaction (RT-qPCR)

Total RNA was extracted from the cells using TRIzol reagent (15596-026, Invitrogen). RNA was reverse-transcribed into cDNA using the PrimeScript™ RT reagent Kit with gDNA Eraser Reverse Transcription Kit (TaKaRa, Madison, WI, USA). qPCR was performed using the Viia™ 7 Real-Time system. PCR products were detected by SYBR^®^ Premix Ex Taq™ (RR420A, TaKaRa). All primers used for qPCR (Table 2) were designed and synthesized by Shanghai GenePharma Company, China. The 2^−ΔΔCt^ method was employed to calculate the relative expression of genes.

**Table 2:** Primer sequence of qPCR.

**Gene**	**Primer sequence**
M-GSDMD-F	ATGCCATCGGCCTTTGAGAAA
M-GSDMD-R	AGGCTGTCCACCGGAATGA
M-GAPDH-F	AGGTCGGTGTGAACGGATTTG
M-GAPDH-R	GGGGTCGTTGATGGCAACA
M-NLRP1-F	CCACTGAGCTACTATGCAGTACA
M-NLRP1-R	ACAACATCTTCACACCACCATC
M-NLRP3-F	ATCAACAGGCGAGACCTCTG
M-NLRP3-R	GTCCTCCTGGCATACCATAGA
M-NLRC4-F	ATCGTCATCACCGTGTGGAG
M-NLRC4-R	GCCAGACTCGCCTTCAATCA
eNOS-F	CGCAAGAGGAAGGAGTCTAGCA
eNOS-R	TCGAGCAAAGGCACAGAAGTGG
GADPDH-F (human)	CATCACTGCCACCCAGAAGACTG
GAPDH-R (human)	ATGCCAGTGAGCTTCCCGTTCAG
Edn1-F	CTACTTCTGCCACCTGGACATC
Edn1-R	CGCACTGACATCTAACTGCCTG
vWF-F	AACAGACGATGGTGGACTCAGC
vWF-R	CGATGGACTCACAGGAGCAAGT

Abbreviations: qPCR: quantitative real-time polymerase chain reaction; GSDMD: Gasdermin D; GAPDH: glyceraldehyde-3-phosphate dehydrogenase; NLRP1: the NOD-like receptor protein 1; NLRP3: the NOD-like receptor protein 3; NLRC4: the NOD-like receptor and inflammasome activator; eNOS: endothelial nitric oxide synthase; Edn1: Endothelin-1; vWF: von Willebrand factor.

### Western blotting analysis

After interventions, cells were lysed at 100°C by water bath method for 10 min. Then, the lysates were centrifuged at 12,000 rpm for 5 min, and the supernatants were collected [18]. Protein samples were separated using 10% sodium dodecyl sulfate-polyacrylamide gel electrophoresis and transferred to PVDF membranes. After blocking with 5% skim milk, the membranes were incubated with goat anti-GFAP (Abcam-ab10062, 1:1000), anti-GSDMD (Abcam-ab215203, 1:1000), anti-caspase 1 (Abcam-ab207802, 1:1000), anti-caspase 4 (Abcam-ab25898, 1:1000), anti-pdcd5 (Abcam-ab83938, 1:1000), anti-action (Abcam-ab6276, 1:5000), anti-GSDME (Abcam-ab215191, 1:1000), anti-pTau (Abcam-ab92676, 1:1000) and anti-Aβ42 (Abcam-ab271968, 1:1000) overnight at 4°C. After three washes with TBST (PBS with 0.05% Tween20), the membranes were incubated with secondary antibodies: anti-mouse IgG horseradish peroxidase-linked antibody (7076) and anti-rabbit IgG horseradish peroxidase-linked antibody (7074) (Cell Signaling Technology, Danvers, MA, USA) for 2 h at 37°C. After five washes with PBST, the protein bands were visualized using an ECL assay kit (Beyotime, Shanghai, China), and analyzed using Image-Pro Plus software (version 6.0, Media Cybernetics, Rockville, MD, USA).

### Enzyme-linked immunosorbent assay (ELISA)

According to the manufacturer's instructions, mouse TNFα ELISA kit, mouse IL-1α ELISA kit, mouse IL-1β ELISA kit, mouse IL-18 ELISA kit, mouse IL-6 ELISA kit, and mouse TGF-β ELISA kit were used to detect the levels of TNF-α, IL-1α, IL-1β, IL-18, IL-6 and TGF-β in the supernatant of cell culture, respectively. All ELISA kits were obtained from Shanghai Senxiong Technology Industry Company (Shanghai, China).

### Immunofluorescence (IF) staining

Astrocytes were injected onto 6-well plates and grown to 70% confluency before stimulation for pyroptosis. The cells were fixed in 4% paraformaldehyde for 15 min. Moreover, 0.1% Trizon X-100 was then used to disrupt the membrane, and the cells were sealed with 5% BSA. The anti-GSDMD monoclonal antibody (Abcam 1:300) was incubated at 4°C overnight. The nuclei are labeled with DAPI. Finally, a confocal microscope was used to view the images.

### Hematoxylin - eosin (H&E) staining

H&E staining was performed according to the method reported in the literature. Brain tissue and aorta tissue were fixed with 4% paraformaldehyde for 24 h, embedded in paraffin, and 4 μm sections were prepared. Sections were stained with H&E. Tissue morphology was observed under a light microscope. The secondary antibody was then added to the sections and incubated for 20 min at 37°C. Staining was visualized using DAB substrate (Mxb Biotech, Fujian, China). Image J was used to analyze the captured photographs.

### Immunohistochemistry

Paraffin-embedded mouse brain tissue and aortic tissue were cut into thin slices. Sections were dewaxed with xylene and rehydrated with gradient ethanol. Then, antigen repair was performed using 0.01 mol/L sodium citrate antigen repair solution (P0081, Beyotime Biotechnology), and endogenous peroxidase activity was blocked using hydrogen peroxide (3.0%). After closure with 10% normal FBS, primary antibodies were added to the sections and incubated overnight at 4°C.

### Statistical analysis

All experiments were repeated at least three times. Data were presented as the mean ± standard error of the mean (SEM). Data were analyzed using two-tailed Student’s *t* test, one-way ANOVA, and two-way ANOVA. GraphPad Prism (version 8.0) was used for data analysis. Statistical significance was set at *p* < 0.05.

## RESULTS

### Aβ42 induced pyroptosis of astrocytes

**Figure 1:**
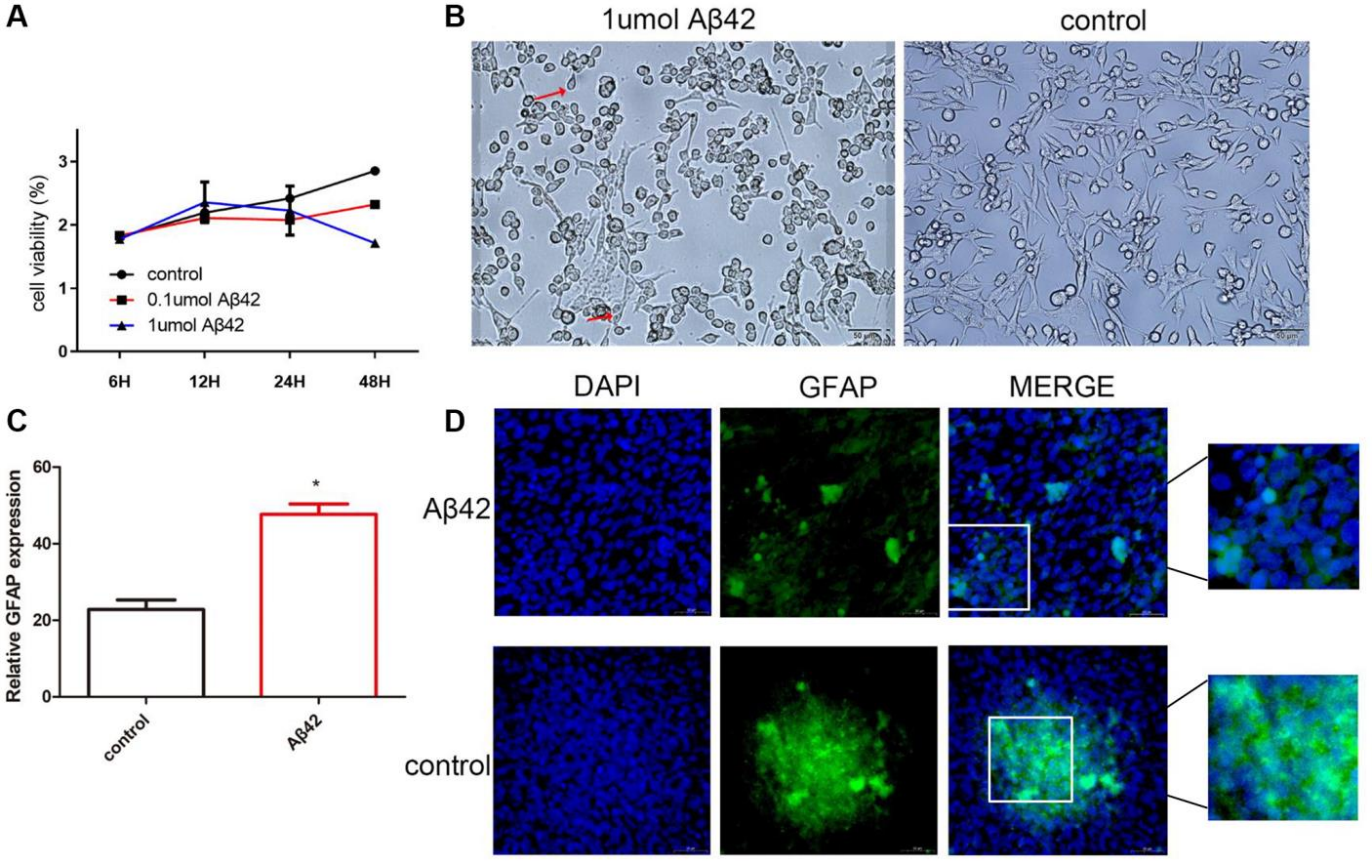
**Aβ42 promotes astrocyte proliferation.** (**A**) CCK-8 assayed the cell proliferation ability of the control cells, 0.1 μmol Aβ42, or 1 μmol Aβ42-treated cells. (**B**) The effect of Aβ42 on astrocyte morphology was observed by scanning electron microscopy. (**C**) Real-time quantitative PCR was used to detect the relative expression of GFAP in the astrocytes treated with 1 μmol Aβ42. ^*^p < 0.05, vs. control group (**D**) Detection of the GFAP expression in astrocytes by immunofluorescence.

To investigate the effect of Aβ42 on astrocytes, astrocytes were treated with different doses of Aβ42 (0.1 μmol, 1 μmol). As shown in Figure 1A, the proliferation capacity of astrocytes significantly increased and peaked within 12 h under the intervention of 1 μmol Aβ42, followed by a gradual decrease in proliferation capacity with time. After 48 h of intervention, compared with the control group, the proliferative capacity of the 1 μmol Aβ42 intervention group was significantly reduced, indicating that 1 μmol Aβ42 inhibited the proliferation of astrocytes after 48 h of intervention. In addition, compared with the control group, astrocyte cell body hypertrophy and swelling and astrocyte protrusion increased and lengthened at 12 h after 1 μmol Aβ42 intervention (Figure 1B). RT-qPCR revealed that the expression of GFAP (biomarker of astrocytes) in the 1 μmol Aβ42 intervention group was significantly higher than that in the 0.1 μmol Aβ42 intervention and control groups (*p* < 0.05, Figure 1C). Immunofluorescence staining further confirmed that after 48 h of intervention, GFAP expression increased and positive cells aggregated into clusters in the 1 μmol Aβ42 intervention group compared with the control group (*p* < 0.001, Figure 1D). Therefore, cells with 1 μmol Aβ42 intervention for 48 h were selected for subsequent analysis.

**Figure 2:**
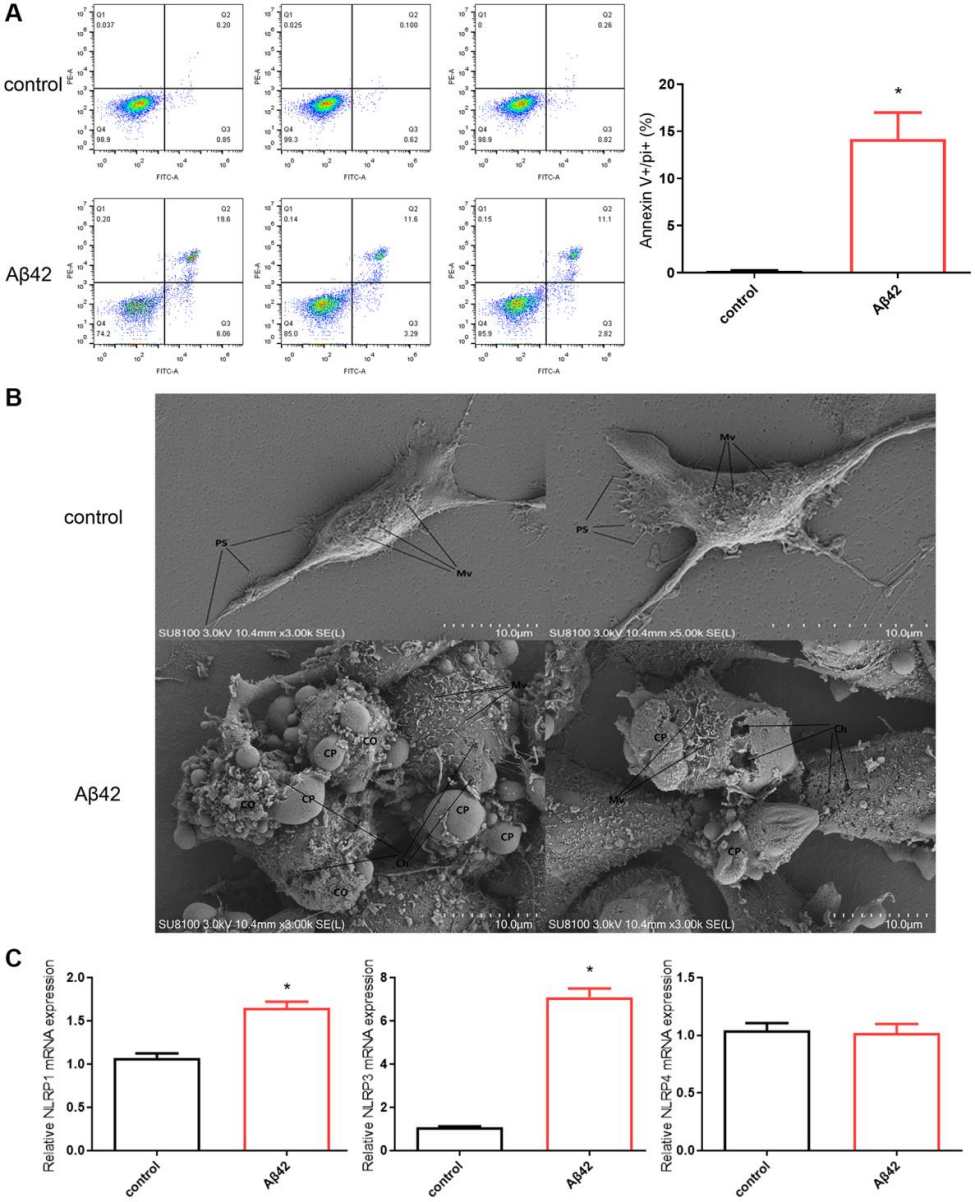
**Aβ42 induces astrocyte pyroptosis.** (**A**) Astrocytes were stimulated with the 1 μmol Aβ42 for 48 h were harvested for PI staining and flow cytometry analysis. (**B**) Scanning electron microscopy was performed to observe astrocyte pyroptosis in the control and Aβ42 groups. (**C**) Real-time quantitative PCR was used to detect the expression of astrocyte pyroptosis-associated inflammasomes (NLRP1, NLRP3, NLRP4) in the control and Aβ42 groups. ^*^p < 0.05 vs. control group. Abbreviations: Mv: microvilli; PS: pseudopods; CP: cell projection; Ch: cell hole; CO: cellular content.

Subsequently, flow cytometric analysis of cells by double-labeling annexin V-FITC and PI revealed a significant increase in double-positive cells after 1 μmol Aβ42 intervention compared to the control group (*p* < 0.01, Figure 2A). SEM results also showed that compared with the control group, the cell damage after 1 μmol Aβ42 intervention was serious, showing obvious signs of pyroptosis, including cell swelling and membrane rupture (Figure 2B). Moreover, RT-qPCR results showed that the expression of pyroptosis-related inflammatory bodies (NLRP1 and NLRP3) was significantly increased in the Aβ42 intervention group compared with the control group (*p* < 0.01, Figure 2C). These results suggest that 1 μmol Aβ42 can induce astrocyte pyroptosis.

### Aβ42 induces astrocyte pyroptosis dependent on the GSDMD/GSDME/Caspase 11/NLRP3 pathway

**Figure 3:**
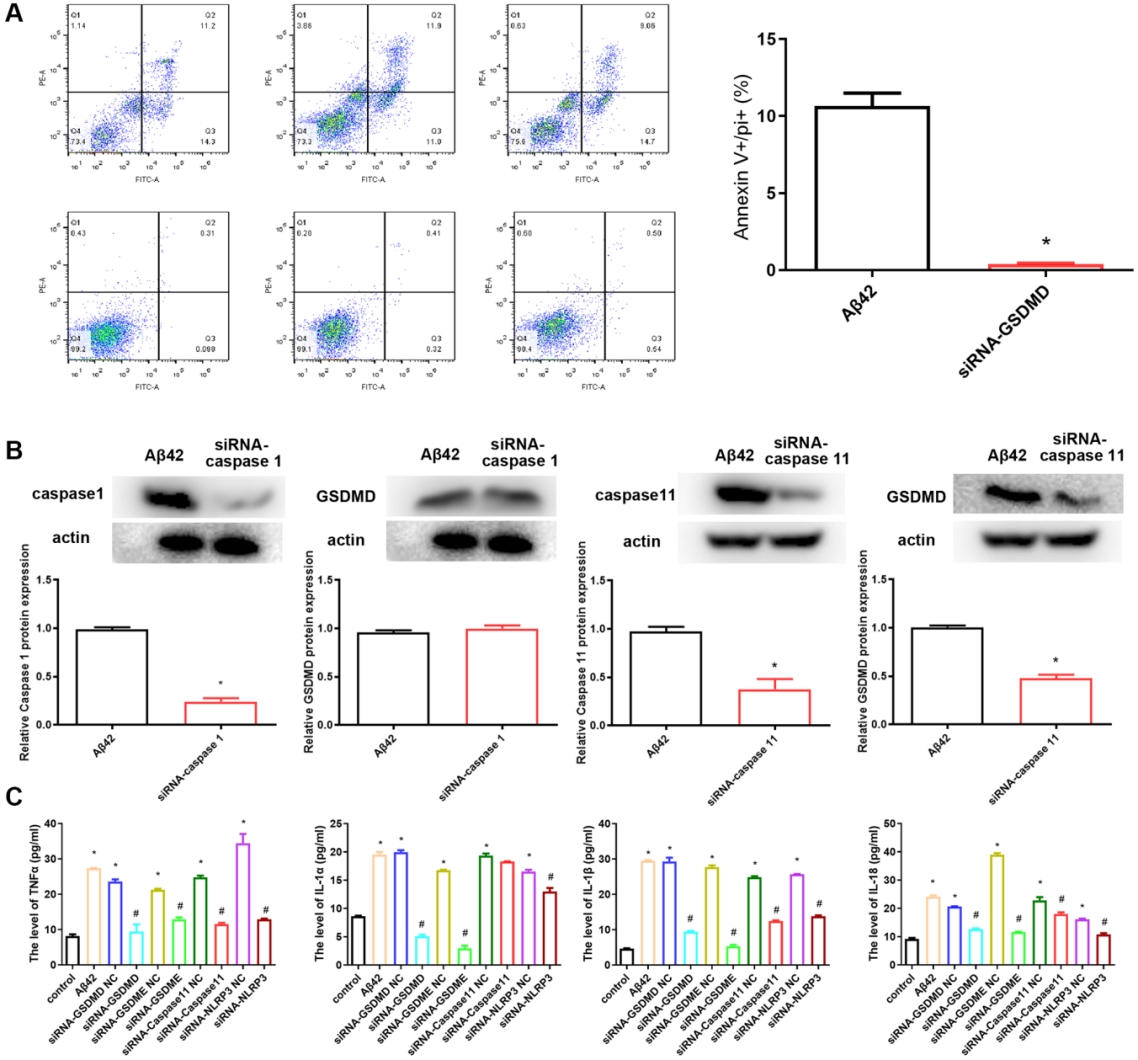
**siRNA-GSDMD inhibits astrocyte pyroptosis.** (**A**) After the knockdown of GSDMD, astrocytes were stimulated with the 1 μmol Aβ42 for 48 h. All cells were harvested for PI staining, and flow cytometry analysis. ^*^p < 0.05 vs. Aβ42 group. (**B**) The protein expression of GSDMD in the Aβ42 and siRNA-caspase 1/11 groups was detected by western blotting. (**C**) Concentrations of TNF-α, IL-1α, IL-1β and IL-18 were measured by enzyme-linked immunosorbent assay. ^*^p < 0.05 vs. Aβ42 group, ^#^*p* < 0.05 vs. Aβ42 group.

To understand the mechanism of pyroptosis induction by Aβ42, we examined the induction of some proinflammatory cytokines, caspases, and gasdermins, which might be upregulated by and/or released from astrocyte pyroptosis. After the knockdown of GSDMD using siRNA and subsequent intervention with Aβ42 for 24 h, the proportion of astrocytes that had undergone pyroptosis was significantly reduced, with a significant difference compared to the Aβ42 intervention group (*p* < 0.01, Figure 3A). Similarly, after the knockdown of caspase 1 and caspase 11 with siRNA and subsequent intervention with Aβ42, the expression level of GSDMD in siRNA-caspase 11 group was significantly decreased compared with the Aβ42 intervention group (*p* < 0.01, Figure 3B). However, the expression level of GSDMD in siRNA-caspase 1 group was not significantly different from that in the Aβ42 intervention group (*p* > 0.05, Figure 3B).

Aβ42-induced astrocyte pyroptosis may be dependent on the GSDMD/GSDME/Caspase 11/NLRP3 pathway; hence, we evaluated its effect on pyroptosis by siRNA knockdown of GSDMD/GSDME/Caspase 11/NLRP3. ELISA results showed that the levels of inflammatory factors (TNFα, IL-1α) and pyroptosis-related factors (IL-1β and IL-18) were significantly increased after Aβ42 intervention compared with the control group, while the levels of TNFα, IL-1α, IL-1β, and IL-18 were significantly reduced after inhibition of GSDMD, GSDME, caspase 11, and NLRP3 expression (Figure 3C).

### Dysfunction of endothelial cells (EC) induced by astrocyte pyroptosis

**Figure 4:**
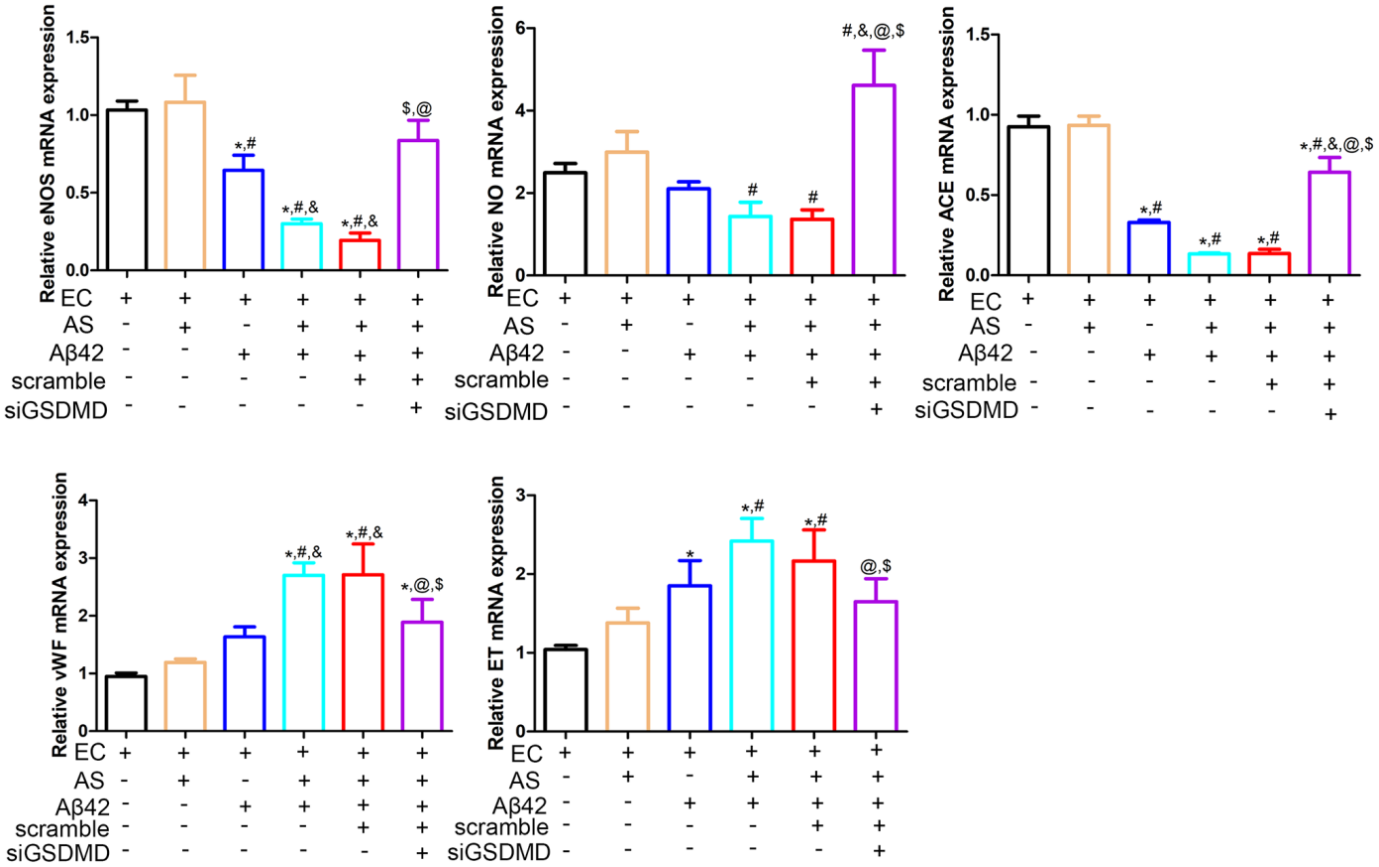
**Real-time quantitative PCR was performed to detect the expression of eNOS, NO, ACE, ET and vWF in endothelial cells.** Abbreviations: EC: endothelial cells; AS: astrocyte. ^*^p < 0.05, vs. EC group, ^#^*p* < 0.05, vs. EC + AS group, ^&^*p* < 0.05, vs. EC + Aβ42 group, ^$^*p* < 0.05, vs. EC + AS + Aβ42 group, ^@^*p* < 0.05, vs. EC + AS + Aβ42 + scramble group.

The structural and functional integrity of the vascular endothelium is fundamental for the normal functioning of the cardiovascular system [19]. In this study, we evaluated the effects of astrocytic pyroptosis on endothelial cell function. There were no significant differences in eNOS, NO, ACE, vWF and ET expression between the endothelial cell only culture group and the endothelial cell and astrocyte co-culture group (*p* > 0.05). In the EC and AS co-culture system, eNOS and NO expression was decreased and ET expression was increased after the addition of 1 μmol Aβ42 intervention, but these changes were reversed by knocking down GSDMD in the astrocytes using siRNA (Figure 4).

### Inhibition of astrocyte pyroptosis can alleviate brain tissue lesions and vascular lesions in APP/PS1 mice

**Figure 5:**
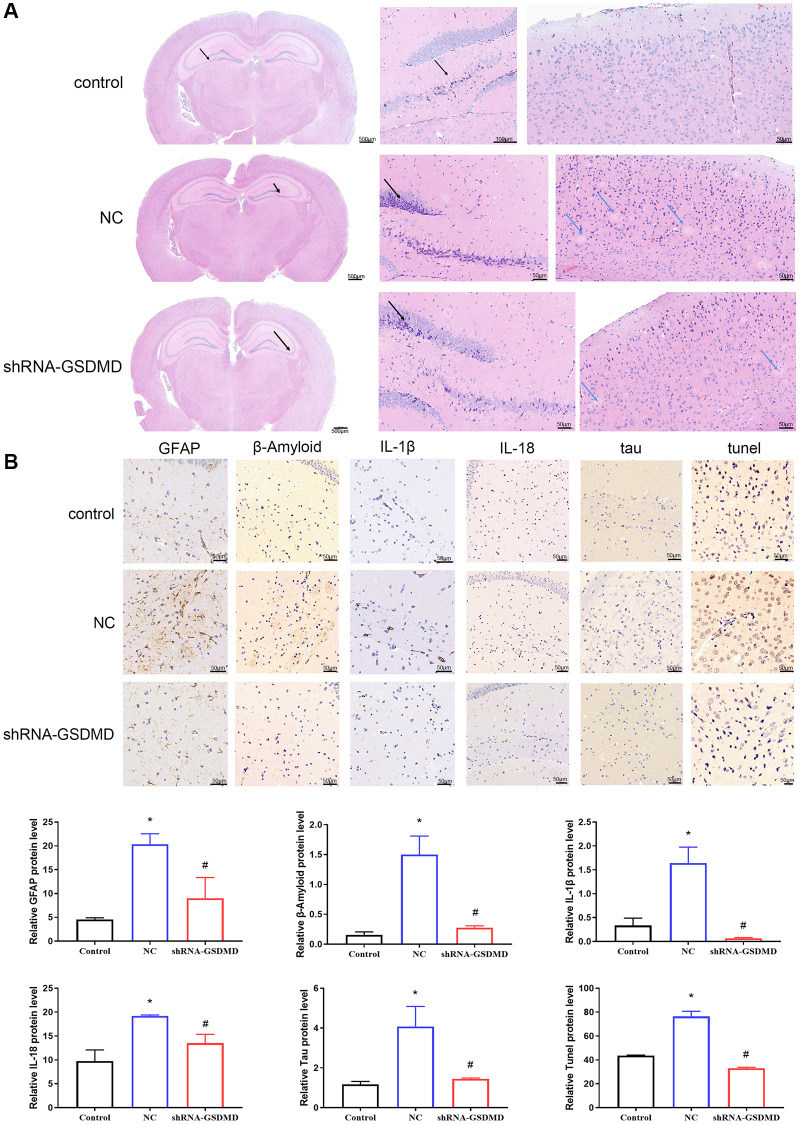
**Inhibition of astrocyte pyroptosis can alleviate brain tissue lesions in APP/PS1 mice.** (**A**) H&E staining of brain tissues. (**B**) Immunohistochemical analysis and protein expression of each group of GFAP, Aβ, IL-1β, IL-18, tau, and tunnel.

H&E staining showed that the cortical neurons were abundant and structurally intact, with no obvious degeneration and necrosis as well as no obvious small plaque formation in the control group. However, the nerve fiber structure disappeared and multiple small plaques formed in the APP/PS1 NC group. Compared to the APP/PS1 model group, these abnormalities were attenuated, with uniform hippocampal nerve fiber staining and no obvious small plaques in the shRNA-GSDMD group (Figure 5A). In addition, immunohistochemical results showed that GFAP, β-Amyloid, IL-1β, IL-18, tau, and tunnel protein expression were significantly higher in hippocampal tissues of NC group mice compared with the control group (*p* < 0.05), and knockdown of GSDMD reversed this alteration (Figure 5B). These results suggest that brain tissue lesions in APP/PS1 mice may be alleviated by GSDMD knockdown to inhibit astrocyte pyroptosis.

**Figure 6:**
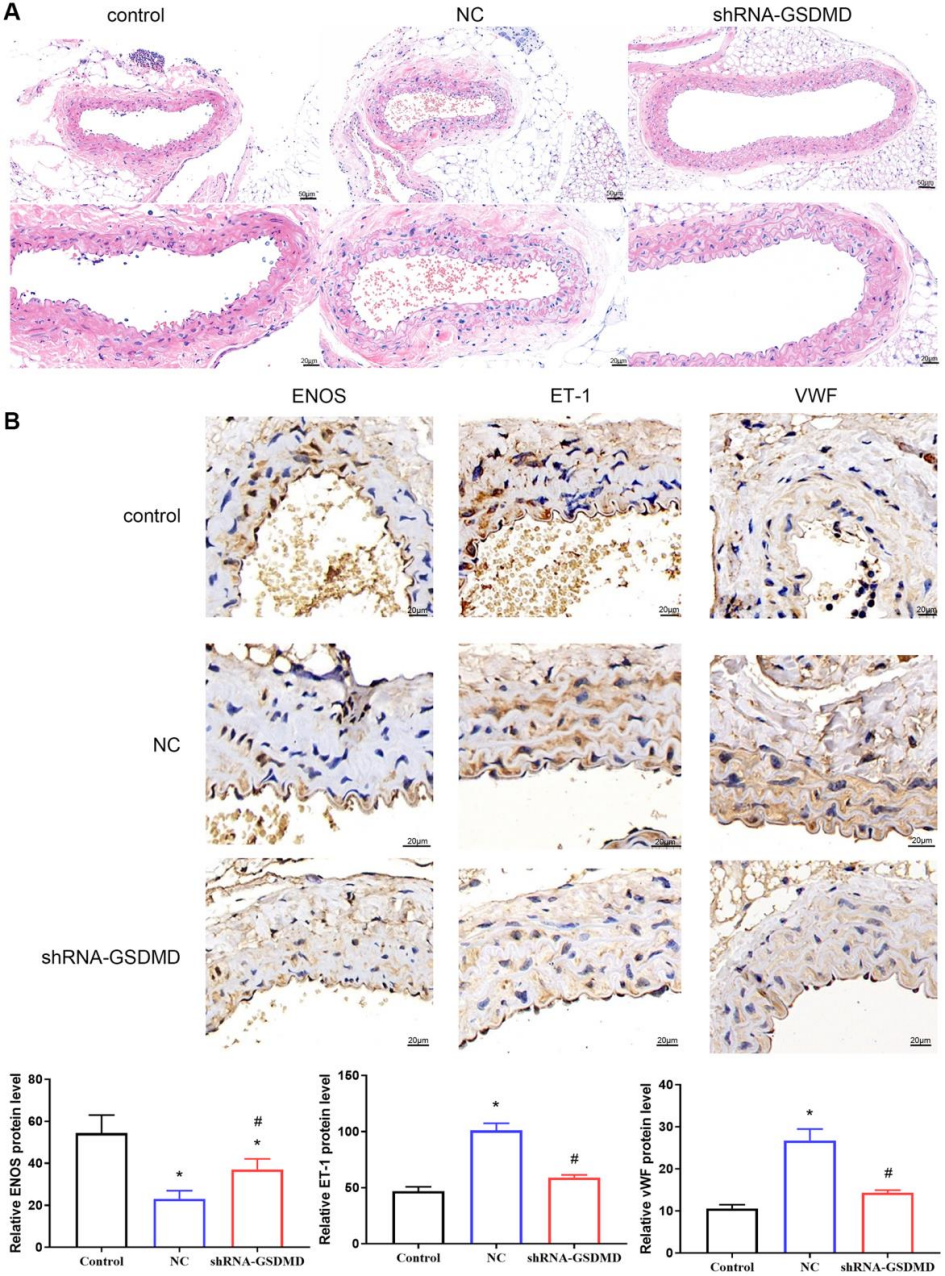
**Inhibition of astrocyte pyroptosis can alleviate vascular lesions in APP/PS1 mice.** (**A**) H&E staining of aortic tissue. (**B**) Immunohistochemical analysis and protein expression of each group of eNOS, ET-1, and vwF.

Similarly, aortic tissue was examined by H&E staining, which revealed an intact endothelium and no significant abnormalities in the outer membrane in the control group. In the NC group, the elastic membrane was loose and tight with segmental straightening and moderate dissolution of collagen fibers in the outer membrane (Figure 6A). The endothelium was intact, and no obvious abnormalities were observed in the outer membrane of the shRNA-GSDMD group. IHC results showed that the expression of Endothelin-1 (ET-1) and von Willebrand factor (vWF) increased, and the expression of eNOS decreased in the NC group compared with the control group. In contrast, the expression of ET-1 and vWF was significantly lower, and the expression of eNOS was significantly higher in the shRNA-GSDMD group than in the NC group, indicating that knockdown of GSDMD could reverse the alterations caused by astrocyte pyroptosis (Figure 6B). These results indicate that aortic tissue lesions in APP/PS1 mice may be alleviated by GSDMD knockdown to inhibit astrocyte pyroptosis, brain tissue damage, and vascular damage.

## DISCUSSION

AD is the most common form of dementia and is characterized by a decline in cognitive function, particularly in memory and judgment. With a rapidly aging population, AD has become a major public health problem. The main pathological features of AD are the presence of extracellular Aβ plaques, intra-neuronal neurogenic fiber tangles consisting mainly of hyperphosphorylated tau and brain atrophy, and increased neuroinflammation in the brain. At the same time, the pathogenic mechanism of Aβ and tau protein can cause changes in the morphology and function of astrocytes, and damage the neuroprotective potential of astrocytes [20]. Previous studies have shown that Aβ can activate glial cells, especially microglia and astrocytes to produce corresponding inflammatory factors, thus promoting the progress of AD [21]. In the present study, we explored the role of Aβ42 on astrocytes and its molecular mechanisms in order to further elucidate the role of astrocyte pyroptosis in AD-related inflammatory responses and its effects on endothelial cells. In this study, we found that Aβ42 induced astrocyte pyroptosis and knockdown of GSDMD inhibited astrocyte pyroptosis, thereby alleviating brain tissue damage and vascular damage in APP/PS1 mice.

Astrocytes are star-shaped cell found in the brain and the spinal cord [22]. They are the most abundant cell type in the central nervous system and are receiving increasing attention for their role in regulating neuronal development and function, metabolism, brain damage, and inflammation [23]. It has been reported that Aβ can induce astrocyte activation, resulting in oxidative stress and cytotoxic inflammatory cytokines and chemokines, which ultimately lead to neuronal death [24, 25]. In an Alzheimer’s disease model with transgenic Tg2576 mice, reactive astrocytes surrounding Aβ plaques expressed high levels of interleukin-1β (IL-1β) [26]. Moreover, there is evidence that astrocytes can express all the components of the NLRP3 inflammasome (NLRP3, ASC and caspase-1), and produce IL-1β in various central nervous system diseases, such as multiple sclerosis, neuromyelitis optica, and cerebral infarction [27]. In a mouse model of traumatic brain injury, cortical astrocytes expressed the components of the NLRP3 inflammasome [28]. Recently, the NLRP1 inflammasome has been identified as a necessary component of the pyroptotic cell death mechanism [29, 30]. It has been reported that NLRP1 is highly expressed in the brain, particularly in pyramidal neurons and oligodendrocytes [31], and this NLRP1 overexpression induces neuronal death [32]. Consistent with previous studies, we found that Aβ42 induced astrocyte pyroptosis, leading to a significant increase in the expression of pyroptosis associated inflammatory bodies (NLRP1, NLRP3).

Pyroptosis is a programmed cell death mediated by GSDMD, which is characterized by swelling, membrane rupture, release of pro-inflammatory cytokines and intracellular contents, and subsequent inflammatory response [33, 34]. Pyroptosis occurs when activated caspase-1 or caspase-4/5/11 cleaves the GSDMD protein, releasing the GSDMD-N subunit that forms membrane pores, which is closely related to the pathogenesis of AD [35]. Previous studies have also demonstrated that caspase-1 activation and subsequent GSDMD cleavage play key roles in pyroptosis and neuroinflammation in CNS diseases, such as cerebral ischemia and AD, and that the inhibition of caspase-1 activation can attenuate brain injury [36–38]. Meanwhile, GSDMD is the executioner of pyroptosis in response to inflammasome activation, is present in CNS microglia and oligodendrocytes, and has been shown to play a critical role in neuroinflammation in the pathogenesis of multiple sclerosis and experimental autoimmune encephalomyelitis [39, 40]. Therefore, inhibition of pyroptosis may be a protective measure for neurons. Han et al. stimulated mouse cortical neurons with Aβ1-42 and found that Aβ1-42 induced neuronal pyroptosis through the caspase-1/GSDMD signaling pathway [41]. Xing et al. found that salidroside reduced pyroptosis by inhibiting caspase-1 activation, GSDMD cleavage and reducing interleukin-1β (IL-1β) release, ultimately reducing the formation of atherosclerotic plaques [42]. In APP/PS1 mice, the simultaneous silencing of NLRP1 and caspase-1 reduced neuronal cell death in the cortex and hippocampus, and improved cognitive impairment [43]. It has been reported that inhibition of GSDMD-mediated pyroptosis can reduce hippocampal neuronal damage [44]. Studies have shown that knocking out NLRP1 or caspase-1 gene in APP/PS1 mice can reduce Aβ deposition in the brain and restore long-term potentiation to near baseline levels [45]. The Yi-Zhi-Fang-Dai formula inhibited neuroglial cell pyroptosis-induced blood-brain barrier disruption and aquaporin 11 depolarization by inhibiting caspase 4/1 activation and GSDMD cleavage, ultimately reducing acute accumulation of Aβ and formation of Aβ1-42 oligomers [46]. Similar to their results, our study found that siRNA-GSDMD and siRNA-caspase 1 inhibited astrocyte scorching caused by Aβ42, mainly in the form of reduced scorching and inhibition of inflammatory factor release. In addition, we found that astrocytic pyroptosis is dependent on the GSDMD/GSDME/caspase 11/NLRP3 pathway.

AD is a common neurodegenerative disease, characterized by β-amyloid (Aβ) plaques, tau protein hyperphosphorylation and neuroinflammation [47]. Aβ is the main pathogenic factor of AD, and its deposition in the interstitial space of neurons activates astrocytes and microglia, leading to the release of a large number of pro-inflammatory mediators, thereby damaging neurons [48, 49]. On the other hand, tau protein is a microtubule-associated protein, involved in maintaining the structure and function of neurons [50]. Tau protein hyperphosphorylation causes it to dissociate from microtubules and form neurofibrillary tangles, further aggravating the progression of AD [51]. In addition, overexpression of IL-1β and IL-18 was found in AD brains [52]. Pyrogenic IL-1β plays a key role in activating AD pathogenic neuroinflammation, contributing to neuronal Aβ generation [53]. In our study, *in vitro* experiments found that Aβ42 caused astrocyte pyroptosis and induced inflammatory secretion of IL-1β and IL-18, thereby aggravating the deposition of Aβ and P-tau. After GSDMD knockdown, APP/PS1 mice showed decreased activation of astrocytes in the hippocampus and cerebral cortex, decreased secretion of Aβ, IL-1β, IL-18, and P-tau, and correspondingly decreased amounts of astrocyte and plaque formation in the brain, which was confirmed *in vivo*. These results suggest that the knockdown of GSDMD could inhibit the activation of astrocytes/expression of inflammatory cytokines, reduce tau hyperphosphorylation, and delay pathological changes in AD.

Furthermore, in *in vitro* experiments, we found that astrocyte pyroptosis can affect EC dysfunction, including decreased levels of the vasodilator NO synthase (eNOS), NO, and ACE, and elevated ET and vwF. After GSDMD knockdown, the expression of ET-1 and vwF in the peripheral aortic intima was reduced, while eNOS was increased, as the intimal injury was reduced. Although the morphology, genetic phenotype, and function of microvascular endothelial cells in various organs are not exactly the same, they regulate vascular contraction and relaxation in similar ways. Therefore, we used aortic endothelial cells in this study [54]. The results of this study showed that AS pyroptosis affects vascular endothelial dysfunction, and that blocking pyroptosis can restore endothelial cell function and alleviate cerebral microcirculation disorders, thereby alleviating the development of AD.

This study had some limitations. Since neurons are the primary cells in the brain, and neuronal death/loss is the major cause of brain dysfunction [55], it is important to test the effect of astrolytic pyroptosis on neurons. We did not conduct this study because of limited experimental conditions and funds. We will consider neurons as the key object of our next research to further explore the role of GSDMD inhibition in neurons and brain injury.

## CONCLUSIONS

In summary, this study found that Aβ42 is dependent on the GSDMD/GSDME/caspase 11/NLRP3 pathway to induce astrocyte pyroptosis. Astrocyte pyroptosis released a large number of inflammatory factors such as TNF-α, IL-1α, IL-1β, and IL-18, destroyed the structure and function of EC, and released a large number of vasoconstricting substances such as ET and vwF. GSDMD knockout reduced the pyroptosis in the Aβ42-treated astrocytes, reduced the release of inflammatory cytokines IL-1β and IL-18, reduced Aβ deposition and tau protein, and thus reduced brain tissue damage and vascular damage in APP/PS1 mice. Our findings provide novel insights into the inflammatory response and pyroptosis in AD, and suggest that GSDMD is a potential therapeutic strategy for patients with AD.
